# Prevalence and determinants of violent disciplinary practices for children in Bangladesh: Evidence from a nationally representative survey

**DOI:** 10.1371/journal.pone.0329017

**Published:** 2025-08-08

**Authors:** Raisa Rashid Mim, Mohammad Shahed Masud

**Affiliations:** Institute of Statistical Research and Training, University of Dhaka, Dhaka, Bangladesh; Universidad Católica Sedes Sapientiae: Universidad Catolica Sedes Sapientiae, PERU

## Abstract

Violent discipline, characterized by the use of physical force resulting in bodily pain, discomfort, or resorting to scolding and emotional abuse to correct children’s misbehavior, has gathered considerable attention due to its negative impact on children’s development. This study aims to identify the prevalence and factors associated with different violent disciplinary practices toward children of 1–14 years age in Bangladesh. Utilizing data from the Multiple Indicator Cluster Surveys (MICS) conducted in 2019, this analysis, based on interviews with parents of 70027 children, focuses on three dependent variables measuring violent disciplinary practices: psychological aggression, physical punishment and any violent discipline. The independent variables considered include the child’s age, sex, type of residence (rural or urban), division, education of mothers, child disability, ethnicity and wealth index. Logistic regression models were employed to find the relationships between these variables and different forms of violent discipline, including psychological aggression, physical punishment and any violent discipline (psychological aggression, physical punishment or both), among children in Bangladesh. The results reveal several significant associations with these disciplinary practices. Younger male children, with functional difficulties, those from urban residence and poorer households, residing in the central and southern regions of the country (Khulna, Chattogram), having primary educated mothers and belonging to the Bengali households are at higher risk of any form of violent discipline including psychological aggression, physical punishment. Significantly, our study unveils a strong correlation between any violent discipline and psychological aggression, highlighting that Bangladeshi parents predominantly employ psychological aggression as their primary mode of disciplinary practice. The findings underscore the importance of activating child protection laws and implementing continuous training programs for parents to promote positive parenting practices. To mitigate the use of violent discipline in Bangladesh, the study suggests focusing on improving parental education levels and addressing economic conditions.

## Introduction

Violent child discipline, specifically encompassing physical and humiliating punishment directed at children, stands as a significant and disconcerting global issue. According to Straus and Mattingly [1], forms of violent discipline are categorized into three primary practices: 1) psychological aggression, 2) physical punishment, and 3) severe physical punishment. In all cultures, parents commonly discipline children to prevent behavioral problems and encourage proper conduct [2,3]. However, some use violent methods, not intending harm but often due to complex emotions like anger and frustration, a lack of understanding of the harm, or unfamiliarity with non-violent alternatives. This reliance on violent discipline, irrespective of its form, raises critical concerns as children subjected to violent discipline often experience emotional and psychological trauma, including anxiety, depression, low self-esteem and physical health consequences as well [4–8]. Violent discipline hinders the development of positive social skills, making it difficult for children to form healthy relationships and effectively communicate, empathize, or resolve conflicts, also negatively affects academic performance leading to disengagement from school and limiting future opportunities [9–11]. Long-term exposure can lead to mental health issues in adulthood, increasing the risk of perpetuating the cycle of violence in future generations [12,13].

Numerous studies have consistently indicated that the prevalence of various violent disciplinary behaviors is significant across several countries [14–19]. Approximately 300 million young children experience psychological aggression and physical punishment by parents and caregivers worldwide [20]. In the UK, 71% of parents use mild physical punishment, while 16% resort to severe punishment [21]. In the USA, 90% of parents use corporal punishment [22]. Around 220.4 million children aged 2-4 years in low and middle-income countries are at risk of aggressive discipline [23]. According to UNICEF 60% of 1-year-olds face daily violent discipline, including physical shaking, with 10% experiencing hitting or slapping [24]. The highest synthesized past-year prevalence rate of any violence in Africa was 80% [25], while in the Middle East and Northern region of Africa (MENA) region, 82-88% of children under five experience violence at home [26].

South Asian countries show similar prevalence rates of violent discipline. In India, nearly half of parents report using severe verbal discipline, and 42% use physical discipline [27]. In Singapore, 80% of children experience at least one physical punishment method across all age groups [28]. In Nepal, eight out of ten children face violent discipline [24]. Research in Sri Lanka shows that 46% of children experience physical punishment at home and 80.4% by teachers in school [29]. In Bangladesh, the Multiple Indicator Cluster Survey (2012) reveals that 82.3% of children experience violent discipline. A survey by Bangladesh Legal Aid and Services Trust (BLAST) and Save the Children (SCI) indicates that physical punishment is seen as a convenient method for instilling discipline in schools (69%), an effective means for proper learning (55%), and necessary for keeping children on the right path (27%) [30]. Another survey highlights low educational results (72%), disobedience (65%), and complaints from others (9%) as reasons for violent discipline [31]. Additionally, it is reported that one in three children experiences beating in school [32].

Research in Bangladesh and different countries has revealed multifaceted factors influencing violence against children in domestic settings. These factors encompass individual dimensions as well as socioeconomic characteristics including age and sex of the child, disability status, place of residence (division, district), educational background of mothers or caregivers and financial status etc. Research indicates that boys are more prone to experience physical punishment, while girls normally experience psychological violence, such as scolding [17,31–34]. However, a study from New Zealand contradicts this trend [35]. Child age is a crucial predictor, with a higher prevalence of physical punishment among younger children while higher levels of aggression among older children [34,36]. But some studies has also found that older children experience harsher physical punishment frequently compared to younger children [16,23,37–39].

Urban areas, lower socioeconomic status such as overcrowded living conditions, frequent family conflicts, lack of social connections, and financial difficulties, specifically low educational level of mothers, and lower living standards, are shown to correlate with high levels of violence disciplinary practices against children [14,17,27,32,40–42]. Other recent study contradicted the results mentioned earlier; it showed an increased rate of violence against children in rural households and households with high economic status [33,43]. Child disability significantly increases the risk of experiencing violent discipline, especially for mentally unstable children [44]. Ethnic variations in the practice of violent discipline are noted in Nepal and Laos [45,46].

Following thorough the review of existing literature and background of the study, it is apparent that although a significant amount of research has been carried out within Bangladesh on the topic, most of them considered corporal punishment in Bangladesh School System. As far as our knowledge, no study in Bangladesh used psychological aggression, physical punishment and any violent discipline all together in single study. This study provides an in-depth analysis for finding the current state of violent disciplinary practices and significant determinants in Bangladesh by utilizing the latest available data from MICS, 2019.

## Methodology

### Data source

This study relied on data primarily acquired from the Bangladesh Multiple Indicator Cluster Survey (MICS), an extensive household survey program accomplished by the Bangladesh Bureau of Statistics (BBS) in partnership with UNICEF. This survey is an integral part of the global MICS initiative, reflecting a collaborative effort to collect comprehensive and reliable information on overall welfare of households and individuals specially children aged 1-17 and women of reproductive age (15-49) in Bangladesh. The MICS 2019 survey took place from January 19, 2019, to June 1, 2019, employing a two-stage sampling approach. The primary sampling unit (PSU) comprised 3,220 units, with a total of 64,400 households sampled. The sampling process involved creating strata based on whether an area was urban or rural in each district. Probability proportional to size (PPS) sampling technique was used, where enumeration areas within each district were obtained based on their size. A proper list of households was obtained and randomly 20 households were sampled from each sampled PSU.

To comprehensively assess the circumstances of children and women nationwide, the study employed five questionnaires. We have only focused on the last two datasets. These include: (a) A questionnaire for children under five that is delivered to the mother (or caregivers) of each child living in the home, (b) A questionnaire for children aged 5-17 years, presented to the mother (or caretaker) of one randomly selected child aged 5-17 years living in the household.

For our study, we restricted the analysis to children aged 1 to 14 years as this is the age range used in the MICS dataset for the violent discipline section. Subsequently, these datasets were merged into one, weighted appropriately, resulting in a final sample size of 70,027 children. The data is available on the MICS website free of cost (https://mics.unicef.org/surveys).

### Outcome variable

This study assessed three types of violent discipline: psychological aggression, physical punishment, and any violent discipline which includes either psychological aggression, physical punishment or both. Thus the outcome variable for this study is the different types of violent discipline practices, as measured by the Parent-Child Conflict Tactics Scale [47]. This scale includes eight distinct behaviors that are categorized under the following types of child discipline:

- Psychological aggression
- Physical punishment
- Any violent discipline

The eight behaviors and their correspondence to the specific type of child discipline are outlined in tab:1.

**Table 1:** Types of violent child discipline behaviors in child discipline module.

Child discipline module	Category
Psychological aggression	Shouting, yelling, or screaming at child
	Calling child dumb, lazy, or another name
Physical punishment	Shaking child
	Spanking, hitting, or slapping child on bottom with bare hand
	Hitting child on the bottom or elsewhere with belt, brush, stick, etc.
	Hitting or slapping child on the hand, arm, or leg
	Hitting or slapping child on the face, head, or ears
	Beating child up as hard as one could
Any violent discipline	Any of the above behaviors

Psychological aggression has two items including shouting, yelling or screaming at child and calling a child lazy. The original items are binary variables with yes and no answers. If a child experience at least one of the mentioned behaviors that is if a child experience either shouting, yelling, screaming, called dumb, or he/she experience both then we code psychological aggression as 1 indicating the presence of aggression, otherwise 0 indicating absence. Physical punishment includes six items. They are whether a child has been shaken, spanked, hit, slapped on different body parts by bare hand or using some hard objects. They are combined together such that if a child experience at least one such behaviours, we code physical punishment as 1, otherwise 0 with yes and no answers. The overall response variable, any violent discipline (1 and 0) is measured based on all mentioned behaviours earlier. It is categorized as 1 if a child experience either psychological aggression, physical punishment or both, otherwise it takes value of 0.

### Independent variable

Based on previous literature, the independent variables were selected. We consider demographic and socioeconomic variables including sex and age of child, area, division, mother’s education, child’s functional difficulties, ethnicity of household head and wealth index. A full description of the variables is presented in tab:2.

**Table 2:** Description of variables.

Variables	Categories	Coding
Child’s sex	Male	1
	Female	2
Child’s age	1-2	1
	3-4	2
	5-9	3
	10-14	4
Mother’s education	Pre-primary or none	1
	Primary	2
	Secondary	3
	Higher secondary+	4
Area	Urban	1
	Rural	2
Division	Barishal	1
	Chattogram	2
	Dhaka	3
	Khulna	4
	Mymensingh	5
	Rajshahi	6
	Rangpur	7
	Sylhet	8
Child’s functional difficulties	Has functional difficulty	1
	Has no functional difficulty	2
Ethnicity of household head	Bengali	1
	Other	2
Wealth index quintile	Poorest	1
	Second	2
	Middle	3
	Fourth	4
	Richest	5

### Statistical analysis

Univariate analyses are performed to provide a clear and organized summary of the variables. Bivariate analyses are used to observe the association of each determinant over the outcomes and the chi-square test assessed the significance of associations. To show correlation among the disciplinary behaviors, we used cramer’s V. For multivariate analysis we performed logistic regression since our response variable is a categorical variable with two categories. In this analysis, we adjusted for potential confounders, including the child’s sex and age, area (urban/rural), division, mother’s education, child’s functional difficulties, ethnicity of the household head, and household wealth index. These variables were selected based on theoretical relevance and prior research. The adjusted odds ratios (aOR) are calculated, reported, and interpreted with a 95% confidence interval for each level of the determinants. The statistical analyses are conducted with the help of the package “svy” in Stata (StataCorp version 14.0) software. For producing the district and division wise maps we used R software and Bangladesh shape file.

## Results

### Descriptive analysis

#### Univariate analysis.

In this study, a sample of 70027 individuals was chosen. tab:3 gives us the frequency and percentage distribution of dependent variables. Approximately 86.33% of the studied sample, reports psychological aggression, 64.59% experience Physical punishment and about 88.84% of individuals reports of experiencing any violent discipline in the month preceding the survey.

**Table 3:** Frequency and percentage distribution of outcome variables.

Outcome variable	Frequency	Percentage (%)
**Psychological aggression**		
Yes	60,452	86.33
No	9,575	13.67
**Physical punishment**		
Yes	45,229	64.59
No	24,798	35.41
**Any violent discipline**		
Yes	62,210	88.84
No	7,817	11.16

A comprehensive snapshot of demographic characteristics and contextual factors related to child discipline is shown in tab:4. The gender distribution is nearly equal, with 50.5% male and 49.5% female children. Age-wise, the dataset exhibits diversity, with a notable presence of children aged 5–9 years (35.6%). Residence is predominantly rural (79.5%), reflecting the study’s broader representation.

Divisional distribution is varied, with significant participation from regions such as Dhaka (23.5%), Chattogram (21.6%), and Rajshahi (11.8%). Maternal education levels span from pre-primary to higher secondary+, indicating educational diversity. A small proportion of children report functional difficulties (7%). Bengali households constitute the majority (98.8%) ethnically. The study covers a spectrum of economic statuses, with balanced representation across wealth quintiles.

**Table 4:** Demographic characteristics of the study population: frequency and percentage distribution.

Characteristic	Frequency	Percentage (%)
**Child’s Sex**		
Male	35,367	50.5
Female	34,660	49.5
**Child’s Age**		
1–2 years	9,053	12.9
3–4 years	9,462	13.5
5–9 years	24,911	35.6
10–14 years	26,601	38.0
**Place of residence**		
Urban	14,364	20.5
Rural	55,663	79.5
**Division**		
Barishal	4,105	5.9
Chattogram	15,101	21.6
Dhaka	16,468	23.5
Khulna	7,073	10.1
Mymenshing	5,436	7.8
Rajshahi	8,228	11.8
Rangpur	7,563	10.8
Sylhet	6,052	8.6
**Mother’s education**		
Pre-primary or none	15,225	21.7
Primary	19,115	27.3
Secondary	19,115	27.3
Higher secondary+	6,948	9.9
**Child’s functional difficulties**		
Has functional difficulty	4,934	7.0
Has no functional difficulty	60,650	86.6
**Ethnicity of household head**		
Bengali	69,172	98.8
Other	855	1.2
**Wealth index quintile**		
Poorest	16,051	22.9
Second	14,674	21.0
Middle	13,269	18.9
Fourth	12,940	18.5
Richest	13,094	18.7

#### Associations among the disciplinary behaviours.

Our next analysis is to understand the relation among the disciplinary behaviors. We used cramer’s V for this purpose. We have explored correlations among different types of violent child discipline. our investigation will focus on computing correlations for the behaviors listed below:

- Child subjected to psychological aggression
- Child subjected to physical punishment
- Child subjected to any violent discipline

**Table 5:** Correlations between types of violent discipline.

	Psychological aggression	Physical punishment	Any violent discipline
**Psychological aggression**	1		
**Physical punishment**	0.4074	1	
**Any violent discipline**	0.887	0.5077	1

From tab:5 we can clearly say that there is moderate correlation (0.407) between psychological aggression and physical punishment, suggesting a visible connection between these two disciplinary approaches. It simply indicates that the two are related in a way that when one is present, the other is more likely to be present too, that is physical punishment and psychological aggression tend to occur together. Notably, there is a very strong correlation between any violent discipline and psychological aggression (0.887), while the correlation with physical punishment is comparatively weaker (0.5077). This is the most crucial finding, that is in the context of disciplining children, psychological aggression plays a more substantial role. In other words, a majority of individuals choose psychological aggression as a primary means of disciplining their children.

#### Bivariate analysis.

Percentage distributions of children age 1-14 years by child disciplining methods experienced during the last one month are represented in tab:6. It reveals some variations between male and female children for disciplining with physical punishment methods. It seems that boys are more likely to experience punishment compared to girls (67.2% vs 61.9%). However, psychological aggression (86.7% vs 86%) and any violent discipline method (89.2% vs 88.5%) did not show any significant differences with the percentages. There are also differences based on child’s age. The prevalence of different violent discipline methods increased with age, peaking at age 3-9 and then fell in the oldest group, 10-14. The prevalence of aggression and any violent discipline is quite similar for age group, 3-4 (90.4 vs 93.6%) and 5-9 (90.3% vs 92.6%), while for physical punishment, the prevalence seems to decrease exponentially (3-4 age group: 81% vs 5-9 age group: 74.5%) and it becomes lowest in the 10-14 age group (49.2%).

**Table 6:** Percentage of children age 1-14 years by child disciplining methods experienced during the last one month, Bangladesh, 2019.

Characteristic	Psychological aggression (%)	Physical punishment (%)	Any Violent discipline (%)
Total	86.3	64.6	88.8
**Sex**		***	
Male	86.7	67.2	89.2
Female	86.0	61.9	88.5
**Age**	***	***	***
1-2 years	78.5	65.5	82.8
3-4 years	90.4	81.0	93.6
5-9 years	90.3	74.5	92.6
10-14 years	83.8	49.2	85.7
**Area**			
Urban	86.8	64.6	89.3
Rural	86.2	64.6	88.7
**Division**	***	***	***
Barishal	76.1	55.5	79.7
Chattogram	87.2	66.5	90.2
Dhaka	86.6	67.0	89.0
Khulna	89.7	66.7	91.9
Mymensingh	85.0	63.4	86.9
Rajshahi	86.8	59.0	88.7
Rangpur	87.7	64.0	89.4
Sylhet	85.1	66.2	89.0
**Mother’s education**	***	***	***
Pre-primary or none	84.8	60.4	87.1
Primary	87.6	66.4	90.0
Secondary	87.1	66.9	89.7
Higher secondary+	82.7	59.3	85.6
**Child’s functional difficulties (age 2-14 years)**			
Has functional difficulty	88.8	65.6	91.0
Has no functional difficulty	87.1	65.0	89.5
Ethnicity of household head	***		***
Bengali	86.4	64.7	88.9
Other	79.6	57.2	82.7
Wealth index quintile	***	***	***
Poorest	86.5	66.3	89.0
Second	86.9	65.3	89.1
Middle	87.2	66.4	89.5
Fourth	85.8	64.0	88.6
Richest	85.1	60.4	87.9

p  <  0.05; **p < 0.01; ***p < 0.001.

**Fig 1:**
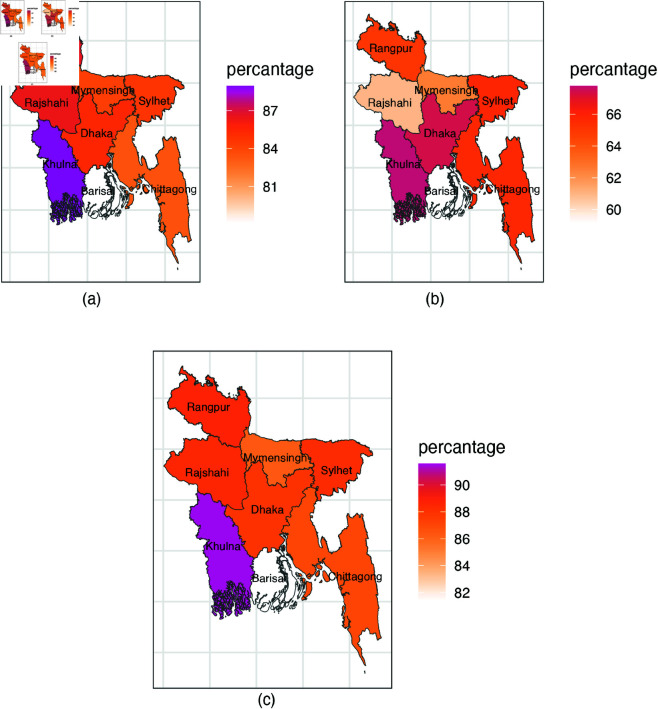
Division wise prevalence of violent disciplinary practices for children (a) psychological aggression (b) physical punishment (c) any violent discipline.

In case of area, both urban and rural areas have similar percentage of prevalence irrespective of the different violent disciplinary behaviors. As for division, it seems that Barishal has the lowest prevalence of psychological aggression (76.1%) and any violent discipline (79.7%), while Khulna has the highest prevalence (89.7% and 91.9%, respectively). Dhaka exhibits the highest prevalence of punishment methods (67%), while Barishal is at the lowest (55.5%). Division-wise prevalences for each violent disciplinary behaviour are shown in Fig 1. Children whose mothers have higher education or college education are less likely to experience any violent discipline practice than children whose mothers had pre-primary or primary education (85.6% vs 87.1% and 85.6% vs 90%). The difference is reflected in both types, psychological aggression( a gap of 5%) and physical punishment( a gap of 6%). Child with functional difficulties are likely to experience psychological aggression rather than physical punishment (88.8% vs 65.6%). Again Bengali households exhibit higher prevalence in violent disciplinary methods compared to other ethnic groups (physical punishment : 64.7% vs 57.2%). Middle class households are likely to experience any violent discipline method than those living in richest households (89.5% vs. 87.9%). Notable differences also exist in psychological aggression (87.2% vs. 85.1%) and physical punishment (66.4% vs. 60.4%) between middle and richest socioeconomic groups.

**Fig 2:**
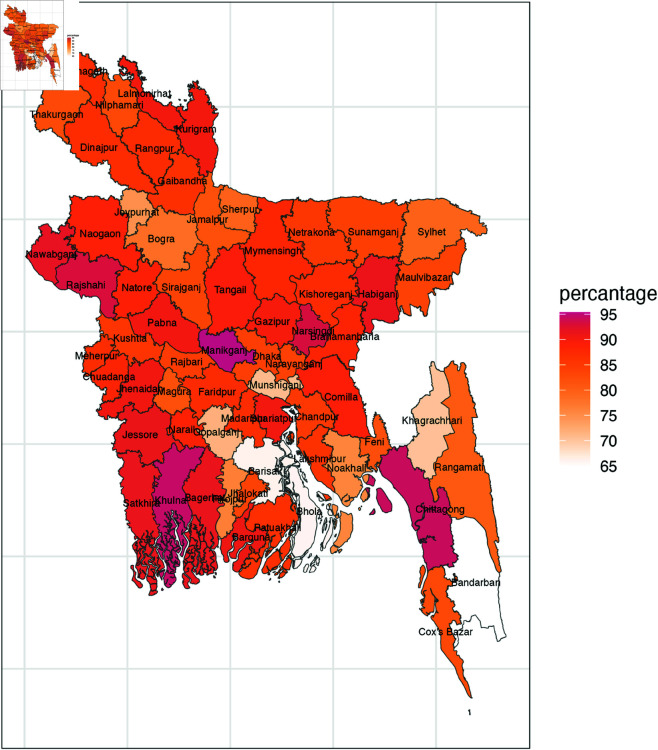
District wise prevalence of psychological aggression.

**Fig 3:**
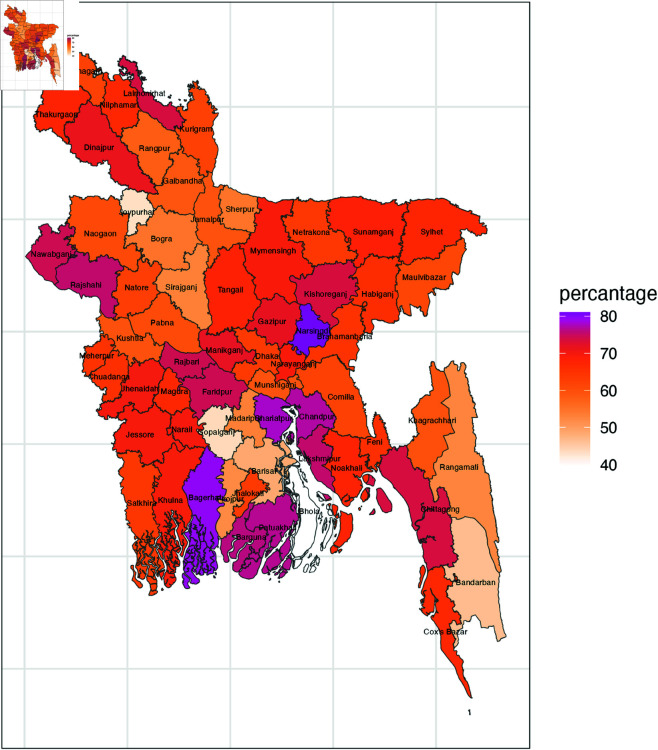
District wise prevalence of physical punishment.

**Fig 4:**
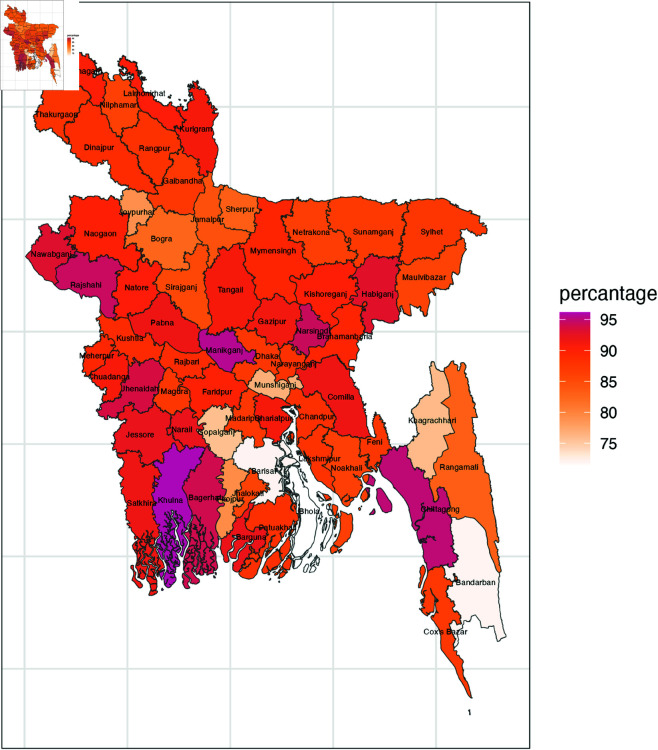
District wise prevalence of any violent discipline.

Categorizing 64 district in a tabular format becomes overly complex, so in Figs 2, 3, and 4 district wise maps based on different forms of violent discipline is presented. Manikganj, Rajshahi, Narsinghdi, Chittagong, and Khulna consistently exhibit the highest levels of psychological aggression, with districts like Habiganj, Comilla, Nawabganj, and others showing moderately high levels of psychological aggression. In terms of physical punishment, Narsinghdi stands out with the highest prevalence, followed by strong prevalence in Bagerhat, Chandpur, and Shariatpur, while Rajbari, Faridpur, and others show moderately high levels. Finally, the overall prevalence of any violent discipline follows the patterns observed for psychological aggression, with districts like Manikganj, Narsinghdi, Khulna, and Bagerhat showing the largest prevalence, while Munshiganj, Khagrachari, and Gopalganj display very low prevalence, and Barishal, Bhola, and Bandarban stand out with the lowest prevalence.

### Logistic regression

For multivariate analysis, we use logistic regression and the results are shown in tab:7. We can see that female children have less odds of any form of violent discipline compared to male children and they are less prone to physical punishment methods compared to psychological aggression (aOR-0.77). Those aged 3-4 years old have 76% more odds of psychological aggression methods compared to 1-2 years old (aOR-1.76). It’s quite same for other forms. While at puberty the odds become less and 10-14 years old children have 5% or 17% less odds for psychological aggression (aOR-0.95) and any violent methods (aOR-0.83). It seems like physical punishment is quite uncommon in puberty’s as they have 70% less odds of punishment method (aOR-0.35).

Residing in rural area and non-Bengali people have less odds of any form of violent discipline method. Rural people have 15% less odds of psychological aggression method (aOR-0.85) compared to urban people while other ethnic groups has 50% less odds (aOR-0.50). Child with functional difficulties has 31% more odds of psychological aggression method (aOR-1.31) while odds for physical punishment method is 17% more (aOR-1.17) and overall they have 39% more odds of any violent discipline . The odds decrease with wealth index as second group shows the highest odds (aOR-0.96) while richest group shows the lowest odds (aOR-0.72) of any form of violence. For the second, middle and fourth group the odds of aggression, physical punishment or any violent discipline is quite similar. While for richest group the odds of psychological aggression is 27% less (aOR-0.72), but for physical punishment the odds is 42% less (aOR-0.58) which is the lowest and overall they have 33% less odds of using any form of violent discipline (aOR-0.67).

Mothers education level is a significant factor. Here we see that mothers with primary education has 31% more odds of psychological aggression method (aOR-1.31) and 23% more odds for physical punishment method (aOR-1.23) compared to no education or pre primary educated mothers. This odds declines as education level rises. Thus mother with higher secondary education have 9% ,12% and 10% less odds for psychological aggression (aOR-0.91), physical punishment (aOR-0.88) and any violent discipline (aOR-0.90) respectively.

**Table 7:** Factors associated with different forms of violent child discipline (results of three binary logistic regression analysis)

Variables	Psychological aggression	Physical punishment	Any violent discipline
	aOR(95% CI)	aOR (95% CI)	aOR (95% CI)
**Gender**			
Male (ref.)			
Female	0.93 (0.89-0.98)**	0.77 (0.74-0.80)***	0.93 (0.88-0.98)**
**Age of child**			
1-2 years old (ref.)			
3-4 years old	1.76 (1.58-1.95)***	1.59 (1.47-1.73)***	2.05 (1.81-2.31)***
5-9 years old	1.70 (1.55-1.86)***	1.06 (0.98-1.14)	1.71 (1.54-1.89)***
10-14 years old	0.95 (0.87-1.04)	0.35 (0.32-0.37)***	0.83 (0.75-0.91)***
**Place of residence**			
Urban (ref.)			
Rural	0.85 (0.80-0.91)***	0.84 (0.80-0.88)***	0.82 (0.76-0.88)***
**Division**			
Dhaka (ref.)			
Barisal	0.42 (0.38 0.47)***	0.50 (0.46-0.54)***	0.41 (0.37-0.46)***
Chattogram	1.08 (1.01-1.17)	0.95 (0.90-1.00)	1.20 (1.10-1.30)***
Khulna	1.25 (1.13-1.37)***	0.90 (0.84-0.96)**	1.30 (1.17-1.45)***
Mymensingh	0.75 (0.68-0.83)***	0.72 (0.67-0.78)***	0.70 (0.63-0.78)***
Rajshahi	0.94 (0.87-1.03)	0.57 (0.54-0.61)***	0.89 (0.82-0.98)
Rangpur	1.08 (0.98-1.18)	0.78 (0.74-0.84)***	1.04 (0.94-1.15)
Sylhet	0.85 (0.78-0.93)***	0.90 (0.84-0.97)**	0.97 (0.88-1.08)
**Mother’s education level**			
No education and pre-primary (ref.)			
Primary	1.31 (1.23-1.40)***	1.23 (1.17-1.29)***	1.36 (1.27-1.46)***
Secondary	1.26 (1.18-1.35)***	1.22 (1.17-1.28)***	1.33 (1.24-1.42)***
Higher secondary+	0.91 (0.82-1.00)	0.88 (0.82-0.95)**	0.90 (0.81-1.00)
**Child’s functional difficulties**			
Has no functional difficulty (ref.)			
Has functional difficulty	1.31 (1.20-1.45)***	1.17 (1.10-1.25)***	1.39 (1.25-1.55)***
**Ethnicity of household head**			
Bengali (ref.)			
Other	0.50 (0.41-0.60)***	0.58 (0.49-0.67)***	0.45 (0.37-0.54)***
**Wealth index**			
Poorest (ref.)			
Second group	0.96 (0.89-1.03)	0.92 (0.88-0.97)**	0.92 (0.85-0.99)
Middle group	0.93 (0.86-1)	0.92 (0.87-0.97)**	0.91 (0.83-0.99)
Fourth group	0.80 (0.74-0.87)***	0.78 (0.73-0.82)***	0.79 (0.72-0.86)***
Richest group	0.72 (0.66-0.79)***	0.58 (0.54-0.62)***	0.67 (0.61-0.74)***

p < 0.05; **p < 0.01; ***p < 0.001.

## Discussion

In our study we observe that parents use aggressive approaches irrespective of their child’s gender, maintaining a consistent level of scolding, which aligns with previous studies [48–50]. Furthermore it is also revealed that males are more likely to experience physical punishment methods than the female childs [33,43,45,51,52]. This pattern may be influenced by societal perceptions of gendered behavior. Boys are often seen as tougher, more resilient, and less likely to follow directions, while girls are viewed as naturally obedient and willing to listen. This perception leads to the belief that if non-aggressive methods of correction fail, physical punishment is necessary for boys to “toughen up.”

Our result finds that both physical punishment and psychological aggression decreases with age and overall young children experience any form of violent discipline than the older children.

**Fig 5:**
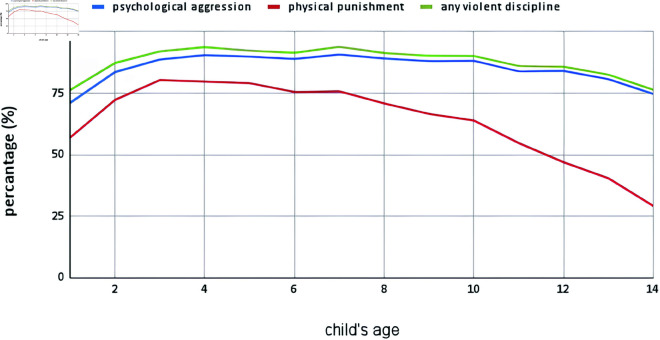
Percentage of different violent disciplinary practices by age.

A closer examination shows that older children receive psychological aggression, while younger children receive physical punishment more, a pattern also observed in prior research [40,42,53–56]. However, this contrasts with some previous findings as well [16,23,57]. The discrepancy may be due to the developmental stage of young children, who, due to limited power, tendency for misbehavior, and communication gaps with parents, are more frequently subjected to physical punishment. In contrast, older children, who are perceived as more mature and capable of managing their responsibilities, often receive non-physical discipline methods like scolding, as parents may feel less need to enforce physical discipline.

This study identifies that residing in rural areas is associated with lower odds of experiencing any form of violent discipline. This may be due to the higher stress and pressures linked to urban living, such as increased population density, social interactions, economic challenges, and competition, which can lead to greater reliance on violent discipline. Despite challenges in rural areas, such as early family formation, lower educational attainment, and larger family sizes, rural households generally use less violent discipline compared to urban areas. Evidence from previous studies supports this finding [16,17,46,58–60]. A study in Vietnam also associates rural areas with experiencing physical punishment [43]. Regionally, in our country, the Southern regions (Chattogram, Khulna) show higher prevalence of violent discipline, while Barishal has the lowest, consistent with Nahar and Amin [32].

We find that parents with higher educational attainment are less likely to employ both psychological and physical aggression, as well as any form of violent discipline, towards children. Moreover, it suggests that educated individuals exhibit a lower prevalence of practicing physical punishment methods compared to psychological aggression methods. Educated individuals may be more aware of alternative disciplinary methods and parenting strategies that focus on positive reinforcement, communication, and understanding rather than resorting to aggression. Additionally, their knowledge about various psychological problems associated with punishing, such as trauma and psychiatric issues, may influence them to opt for non-aggressive means of discipline. The findings are also consistent with other research results [14,27,40,41].

Children with functional difficulties are at a higher risk of experiencing psychological aggression and at moderate risk of physical punishment methods, as reported in both our study and prior research [44]. Lack of parental knowledge or understanding about their specific needs, leads parents to frustration and impatience, resulting in the use of aggressive methods. Additionally, Bengali households are more likely to practice violent discipline than other ethnic groups, with similar ethnic variations observed in studies conducted in Nepal and Laos [45,46].

The study’s results reveal a significant impact of household economic status on violent discipline revealing Second-class households as particularly vulnerable. Wealthier families, with better access to educational resources, parenting support, and information, are more likely to be aware of alternative, non-violent disciplinary methods supported by child development research. This awareness contributes to the creation of nurturing environments actively discouraging aggression. These findings align with previous research [14,17,42,61,62]. Notably, the study acknowledges that despite the expected association between poverty and violence, physical punishment can occur even in wealthy families, as demonstrated by research in Azerbaijan and Vietnam [16,46,63].

From our earlier discussions, it is evident that psychological aggression and any violent discipline approach exhibit a strong correlation, as reflected in the similar results obtained for both. This correlation is underscored by a substantial coefficient of 0.887 between psychological aggression and any violent discipline from the correlation table between types of violent discipline, indicating that aggression is the primary disciplinary method adopted by Bangladesh parents.

Despite global shifts in perspectives on children’s rights and legal restrictions, corporal punishment remains a persistent disciplinary practice in Bangladesh. According to the 2011 ruling of the Supreme Court of Bangladesh, corporal punishment is unlawful in schools [64]. Additionally, the government issued circulars and guidelines in 2010 and 2011 to prohibit corporal punishment in educational institutions [65]. However, these legal measures have not been fully effective in eliminating the practice, as cultural norms continue to uphold corporal punishment as a socially acceptable and effective means of discipline [66,67]. Local studies, such as [68,69], have documented that many parents believe physical discipline is essential for instilling obedience and respect. The belief that “spare the rod, spoil the child” resonates deeply within these cultural contexts, where corporal punishment is considered not only a legitimate form of correction but also an expression of parental love and care. This belief is reinforced by long-standing traditions and social norms, which often perceive physical punishment as a necessary step in teaching respect, obedience, and moral values. Such cultural norms can perpetuate the justification for using physical discipline, as it is viewed as a means to shape children’s behavior and prepare them for adulthood.

Bangladesh is a developing country, positioned between not being excessively impoverished to rely on physical punishment methods and not being affluent enough for widespread adoption of positive parenting practices. Recent improvements in education and literacy, as noted in the 2022 Household Income and Expenditure Survey (HIES) and the functional literacy rate rising to 74.10% in 2023, support the shift towards using psychological aggression over more severe physical punishment methods in disciplinary practices. It highlights a potential evolution in parenting styles that balances cultural beliefs with emerging educational advancements.

## Strengths and limitations

This study’s strength is its ability to generate valuable information regarding the potential factors of different forms of violent child discipline in Bangladesh. Also, this study used a nationally represented dataset that increases reliability by reducing the effect of possible errors. Despite being a nationally representative dataset, the prevalence of severe disciplinary practices may suffer from underestimation since the questions about the disciplinary practices were answered by mothers or other caregivers looking after the children but not the children themselves. The under-reporting may also result from the short reference period (one month before the survey). Moreover, the study is based on available survey data, so it is not possible to include other variables that may affect a child’s risk of violence. Finally, cross-sectional data limited our analysis only to identify the determinants, not the causes of the act, since studying the causal relation- ships requires panel data.

## Conclusion

Violent discipline continues to be a significant issue of worry in the country. Ensuring protection from all types of violence is a basic entitlement outlined in the UN Convention on the Rights of the Child [70]. Therefore, SDG target 16.2 strives to eradicate abuse, exploitation, trafficking, and all forms of violence by 2030. Bangladesh is making progress toward this goal, by ensuring enforcement of child protection laws that explicitly prohibit violent discipline in all settings, including homes and schools [64].

Our study highlights an association between parental violence and factors such as low education levels and economic hardship. Therefore, there is a vital need to initiate programs that generate employment opportunities and enhance financial status as well as the living conditions of families. Simultaneously, continuous efforts are essential to increase women’s educational opportunities. These initiatives should particularly target parents who has positive attitude towards different violent disciplinary practices, those residing in rural areas of Bangladesh, and families facing economic hardship, particularly mothers with limited education. Also, continuous communication, utilizing workshops, seminars, educational materials and education campaigns are vital to promote positive and non-violent disciplinary methods. Specialized training programs for parents, with a focus on positive reinforcement and non-violent communication, contribute significantly to fostering a safer learning environment in home.
